# iTAP: integrated transcriptomics and phenotype database for stress response of *Escherichia coli* and *Saccharomyces cerevisiae*

**DOI:** 10.1186/s13104-015-1759-7

**Published:** 2015-12-12

**Authors:** Niveda Sundararaman, Christine Ash, Weihua Guo, Rebecca Button, Jugroop Singh, Xueyang Feng

**Affiliations:** Department of Biological Systems Engineering, Virginia Tech, Blacksburg, VA 24061 USA; Commonwealth Governor’s School, Fredericksburg, VA 22407 USA

**Keywords:** Open source, Transcriptomics–phenotype correlation, Yeast, *Escherichia coli*

## Abstract

**Background:**

Organisms are subject to various stress conditions, which affect both the organism’s gene expression and phenotype. It is critical to understand microbial responses to stress conditions and uncover the underlying molecular mechanisms. To this end, it is necessary to build a database that collects transcriptomics and phenotypic data of microbes growing under various stress factors for in-depth systems biology analysis. Despite of numerous databases that collect gene expression profiles, to our best knowledge, there are few, if any, databases that collect both transcriptomics and phenotype data simultaneously. In light of this, we have developed an open source, web-based database, namely integrated transcriptomics and phenotype (iTAP) database, that records and links the transcriptomics and phenotype data for two model microorganisms, *Escherichia coli* and *Saccharomyces cerevisiae* in response to exposure of various stress conditions.

**Results:**

To collect the data, we chose relevant research papers from the PubMed database containing all the necessary information for data curation including experimental conditions, transcriptomics data, and phenotype data. The transcriptomics data, including the *p* value and fold change, were obtained through the comparison of test strains against control strains using Gene Expression Omnibus’s GEO2R analyzer. The phenotype data, including the cell growth rate and the productivity, volumetric rate, and mass-based yield of byproducts, were calculated independently from charts or graphs within the reference papers. Since the phenotype data was never reported in a standardized format, the curation of correlated transcriptomics–phenotype datasets became extremely tedious and time-consuming. Despite the challenges, till now, we successfully correlated 57 and 143 datasets of transcriptomics and phenotype for *E. coli* and *S. cerevisiae*, respectively, and applied a regression model within the iTAP database to accurately predict over 93 and 73 % of the growth rates of *E. coli* and *S. cerevisiae*, respectively, directly from the transcriptomics data.

**Conclusion:**

This is the first time that transcriptomics and phenotype data are categorized and correlated in an open-source database. This allows biologists to access the database and utilize it to predict the phenotype of microorganisms from their transcriptomics data. The iTAP database is freely available at https://sites.google.com/a/vt.edu/biomolecular-engineering-lab/software.

## Background

Microorganisms face numerous stress conditions [1–3], such as oxidative stress [4–6], weak organic acid stress [7–9], nutrient limitation [10, 11], and environment fluctuation [12]. These stresses, both biotic and abiotic, occur throughout nature and comprise the ecology of the system [1, 13, 14]. Each stress condition elicits a microbial response to adapt to the unfavorable environmental conditions [15–17]. The provoked responses of microorganisms alter the current ecosystem in which they live and affect the other organisms as well [16, 18]. Such microbial responses could be recreated in laboratories and allow for a deeper understanding of the correlation between gene expressions and phenotypes [4, 7, 12]. Of particular interests to systems biologists, uncovering the correlation between transcriptomics and phenotype could identify ‘genetic markers’ that are primarily responsible for the occurrence of a particular phenotype within a species [6–8]. This would help determine the genetic causations of certain phenotypes across strains. With the genetic markers identified, the phenotype of strains could possibly be predicted from its transcriptomics data directly, which has great potentials in biochemical, ecological, biomedical, and environmental applications [19].

The first step towards uncovering correlations between the transcriptomics and phenotype of various microorganisms is to collect curated and coupled transcriptomics–phenotype datasets for various microorganisms. Currently, there are multiple popular databases such as the Gene Expression Omnibus (GEO) [20–22], the European Bioinformatics Institute (EBI) [23], and Many Microbe Microarrays Database (M3D) [24] that contain gene expression data. Data submitted to these databases is mostly meta-data on transcriptomics analysis, which include experimental conditions and the global gene expressions measured by either microarray [20, 25] or RNAseq analysis [26]. Comprised of over a million samples, these databases allow the analysis of large quantities of transcriptomics data; however, these databases lack the phenotypic data associated with these genotypes. Therefore, although thousands of data series and datasets are enabled for users to query for gene expression analysis, those datasets cannot provide the details about the phenotype such as cell growth rate, and hence, have limited applications in elucidating the correlations between transcriptomics and phenotype of microorganisms.

In this study, we developed an integrated transcriptomics and phenotype (iTAP) database that contained the correlated transcriptomics and phenotype datasets by collecting research articles that reported both types of data during its creation and curating the phenotype data with a standardized format. In general, we collected the transcriptomics data from GEO to provide *p* values and fold changes for each of the genes in *Escherichia coli* and *Saccharomyces cerevisiae* by comparing the gene expression of various strains against a reference strain as indicated in the corresponding publication. In parallel, we collected phenotype data associated with the transcriptomics data and numerically represented them as growth rates, productivity, volumetric rates, and mass-based yield of byproducts. The iTAP database also contained the experimental conditions and stress factors that the strains were subjected to. So far, we have collected, respectively, 143 and 57 datasets for *S. cerevisiae* and *E. coli*. Additionally, we demonstrated that it was feasible to use the correlated transcriptomics–phenotype datasets within the iTAP database to accurately predict cell growth rates for both *S. cerevisiae* and *E. coli* in a proof-of-concept study. Collecting this data proved to be strenuous and time-consuming, which limited the fast scale-up of the iTAP database. As the first of its kind, the iTAP database was able to identify the genetic markers, and potentially, guide synthetic biologists to rationally modify the microbial phenotypes by suppression or overexpression of genes of interests.

## Implementation

### Data collection and curation

**Fig. 1:**
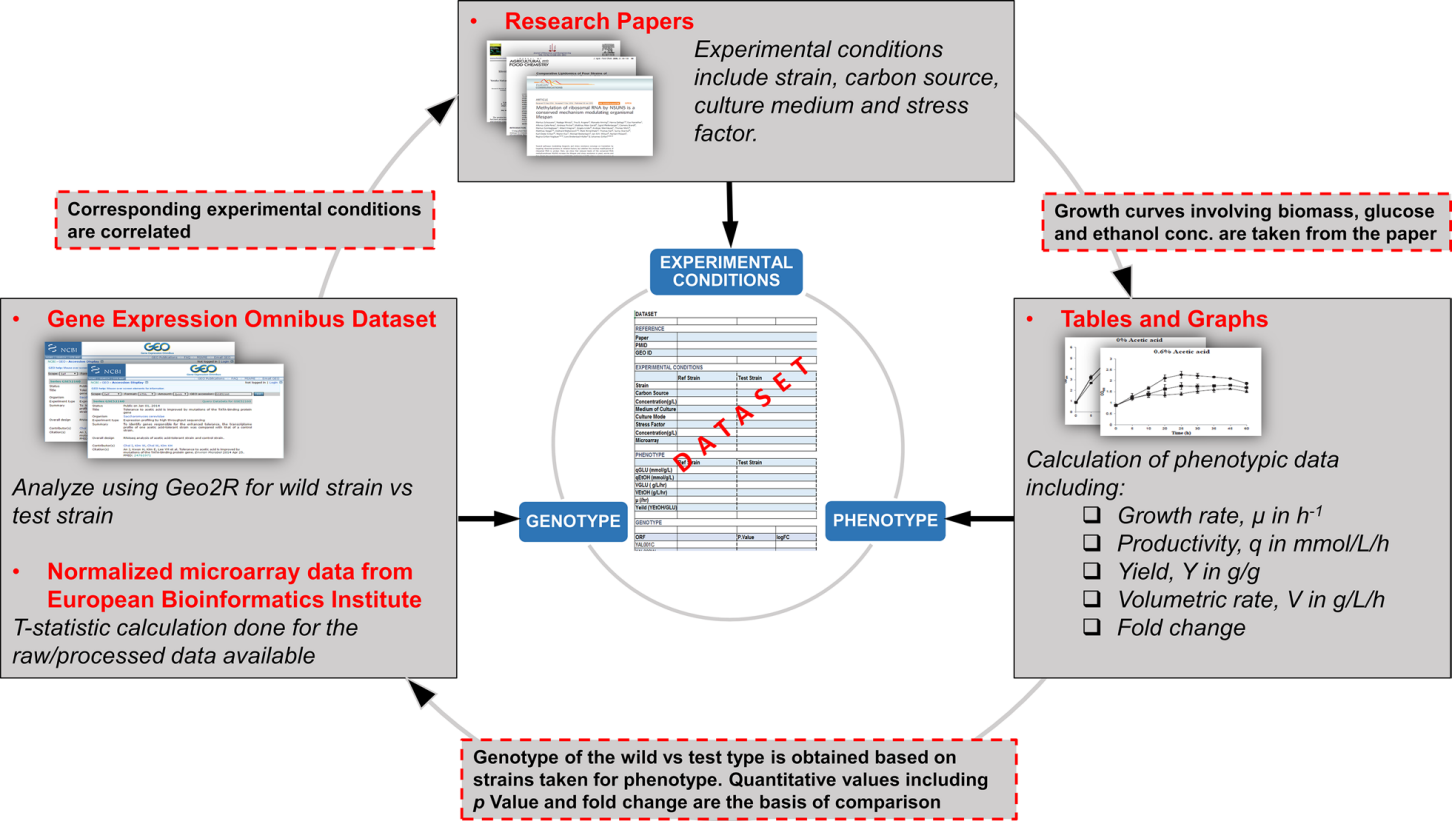
Architecture of the iTAP database

As shown in Fig. 1, each of the dataseries in the database contained experimental conditions, transcriptomics data, and phenotype values of a microorganism, which were obtained from relevant research papers from the PubMed database. To ensure that both transcriptomics and phenotypic data were available in each of the dataseries, only papers that included all of the necessary details mentioned above were chosen to be included within the iTAP database.

**Fig. 2:**
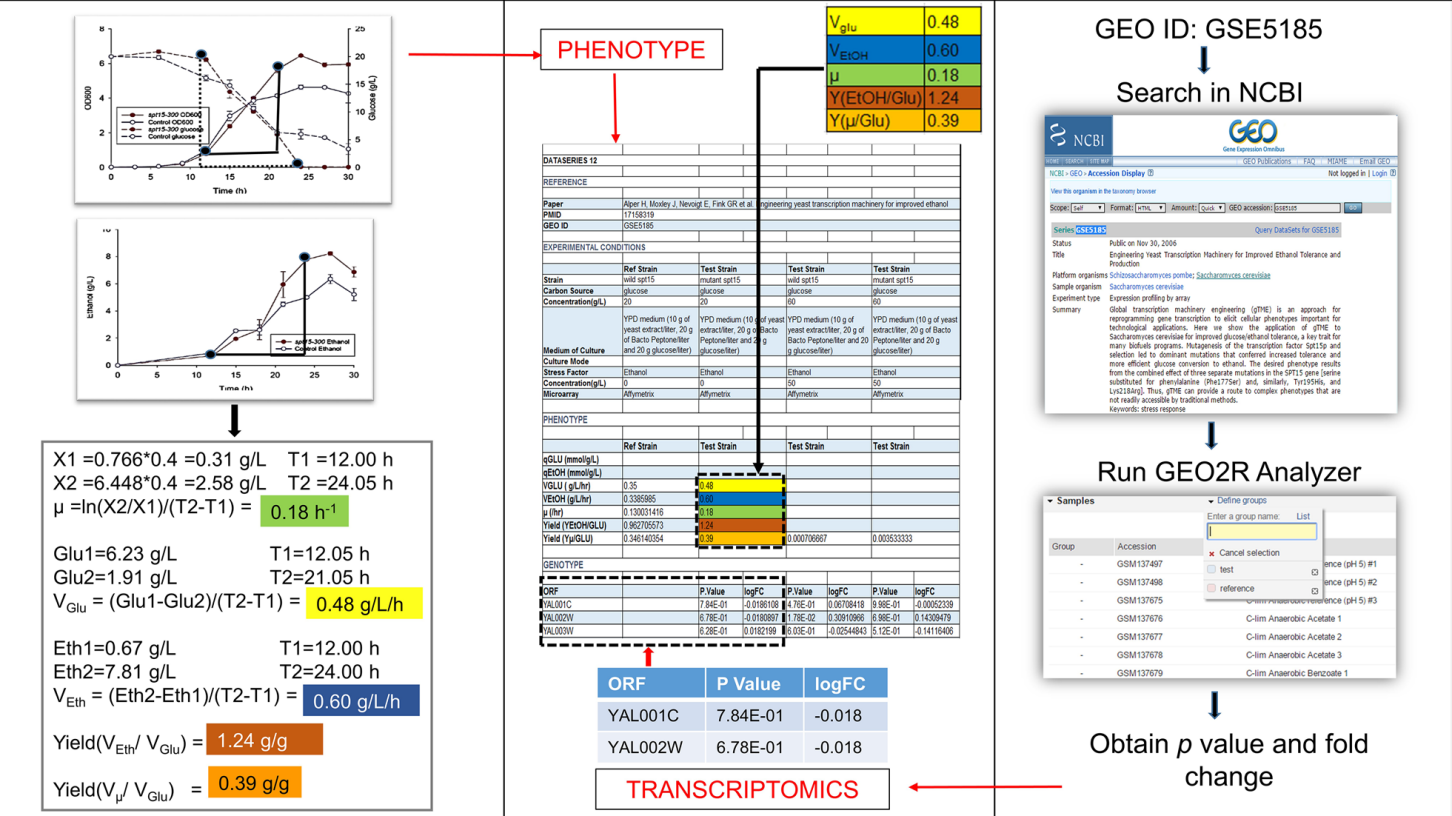
Example of collecting the correlated transcriptomics–phenotype datasets

In general, the experimental conditions were obtained directly from the chosen research papers, including information regarding the strain name, carbon source, culture medium, stress factor, and concentration of the stress factor. The transcriptomics data was collected as the gene expression levels of a test strain subjected to a particular stress condition when compared against a reference strain, and was recorded with *p* values and fold changes. Such data was obtained from the GEO database. The GEO database software tool, GEO2R analyzer [27], was used to compare the gene expression levels of strains facing stress conditions to the reference strain (Fig. 2). After choosing the test and reference strains and running the software, we obtained the corresponding gene expression levels, including *p* value and fold change. It is worth noticing that GEO, EBI and M3D share a lot of transcriptomics data that are exactly the same. Therefore, the same transcriptomics results can be generated when using EBI or M3D. The phenotype data was collected quantitatively as growth rates, productivity, volumetric rates, and mass-based yield of byproducts. Such data was obtained from different charts and graphs from the chosen research papers and was calculated independently. For example, all of the growth rates were calculated directly from the biomass data (e.g., dry cell weights at various time points) in the selected publications while the other phenotype data (e.g. the byproduct rates) was not used for calculating growth rates. Specifically, the phenotype data was calculated as:

1 $${\text{Productivity}}\, ( {\text{mmol/g/h)}}\,{ = }\frac{{{\text{Initial}}\,{\text{concentration}}\,{\text{of}}\,{\text{product}} - {\text{Final}}\,{\text{concentration}}\,{\text{of}}\,{\text{product }}\,\left( {{{\text{mmol}} \mathord{\left/ {\vphantom {{\text{mmol}} {\text{L}}}} \right. \kern-0pt} {\text{L}}}} \right)}}{{\left( {{\text{Final}}\,{\text{time}} - {\text{Initial}}\,{\text{time,}}\,{\text{h}}} \right) \, \times \,{\text{Initial biomass }}\left( {{{\text{g}} \mathord{\left/ {\vphantom {{\text{g}} {\text{L}}}} \right. \kern-0pt} {\text{L}}}} \right)}}$$

where biomass was assumed as: 1 OD = 0.4 g/L;

2 $${\text{Growth}}\,{\text{rate}}\,\left( {{\text{h}}^{ - 1} } \right) = \frac{{\ln \left( {\frac{{{\text{Final}}\,{\text{biomass}}}}{{{\text{Initial}}\,{\text{biomass}}}}} \right)}}{{{\text{Final}}\,{\text{time}} - {\text{Initial}}\,{\text{time}}\,\left( {\text{h}} \right)}}$$

3 $${\text{Volumetric rate}}\,\, ( {\text{g/L/h) = }}\frac{{ {\text{Initial concentration of product}} - \,{\text{Final concentration of product }}\, ( {\text{g/L)}}}}{{{\text{Final time }} - {\text{Initial time (h)}}}}$$

4 $${\text{Yield }}\,({\text{g/g}}) \;{ = }\;\frac{{{\text{Volumetric rate of product (e}} . {\text{g}} . , {\text{ethanol)}}}}{{{\text{Volumetreic rate of carbon source (e}} . {\text{g}} . , {\text{glucose)}}}}$$

We directly used the data when the desired phenotype data was reported in the selected publications. Otherwise, we utilized Plot Digitizer software [28] for graphs within the publication to obtain values of specific points depicting the rate of consumption of glucose, rate of production of products, and growth curves of different strains. All of the data, both the raw data collected from the research papers and the standardized data we calculated, were reported in the iTAP database. It is worth noticing that the majority of the phenotype data collected in the iTAP database were growth related, which is one aspect of the composite of observable characteristics of *E. coli* or *S. cerevisiae*.

### Data distribution

**Fig. 3:**
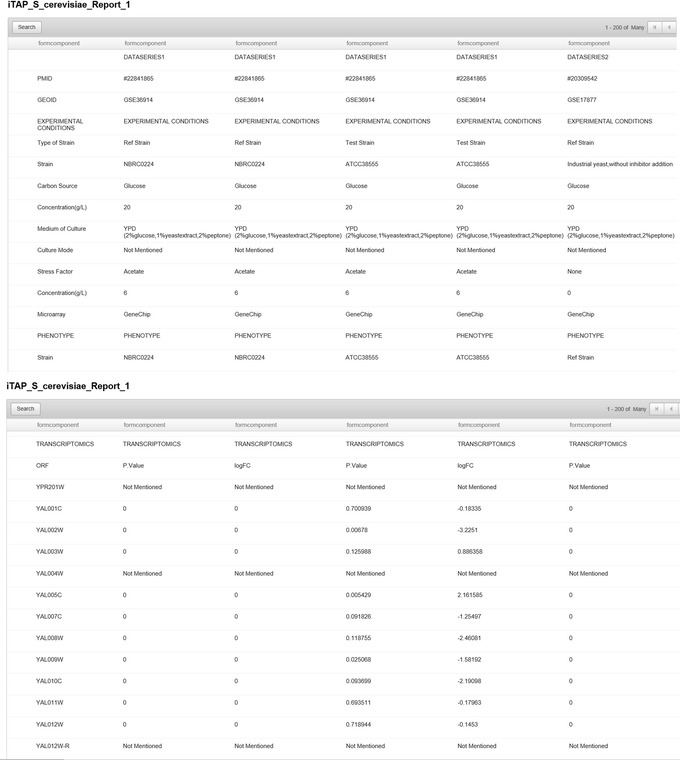
Screenshot of the iTAP database

The iTAP database is an open source, web-based database that is freely available for use (https://sites.google.com/a/vt.edu/biomolecular-engineering-lab/software). It was developed based on Zoho Creator, an online database software that offers the data collection, cloud storage, data backup, and basic data analysis to present all the information in an efficient, user-friendly manner, as shown in Fig. 3. Advanced search with various logic symbols was available for each dataset, allowing users to find their required information efficiently. In addition, user could sort and group one or multiple dataset(s), and browse and print each dataset with different kinds of information by using the “Print” or “View Record” option to output the database. Users were also able to access the real-time data in mobile apps and download any datasets within iTAP as .csv files.

## Results and discussion

**Fig. 4:**
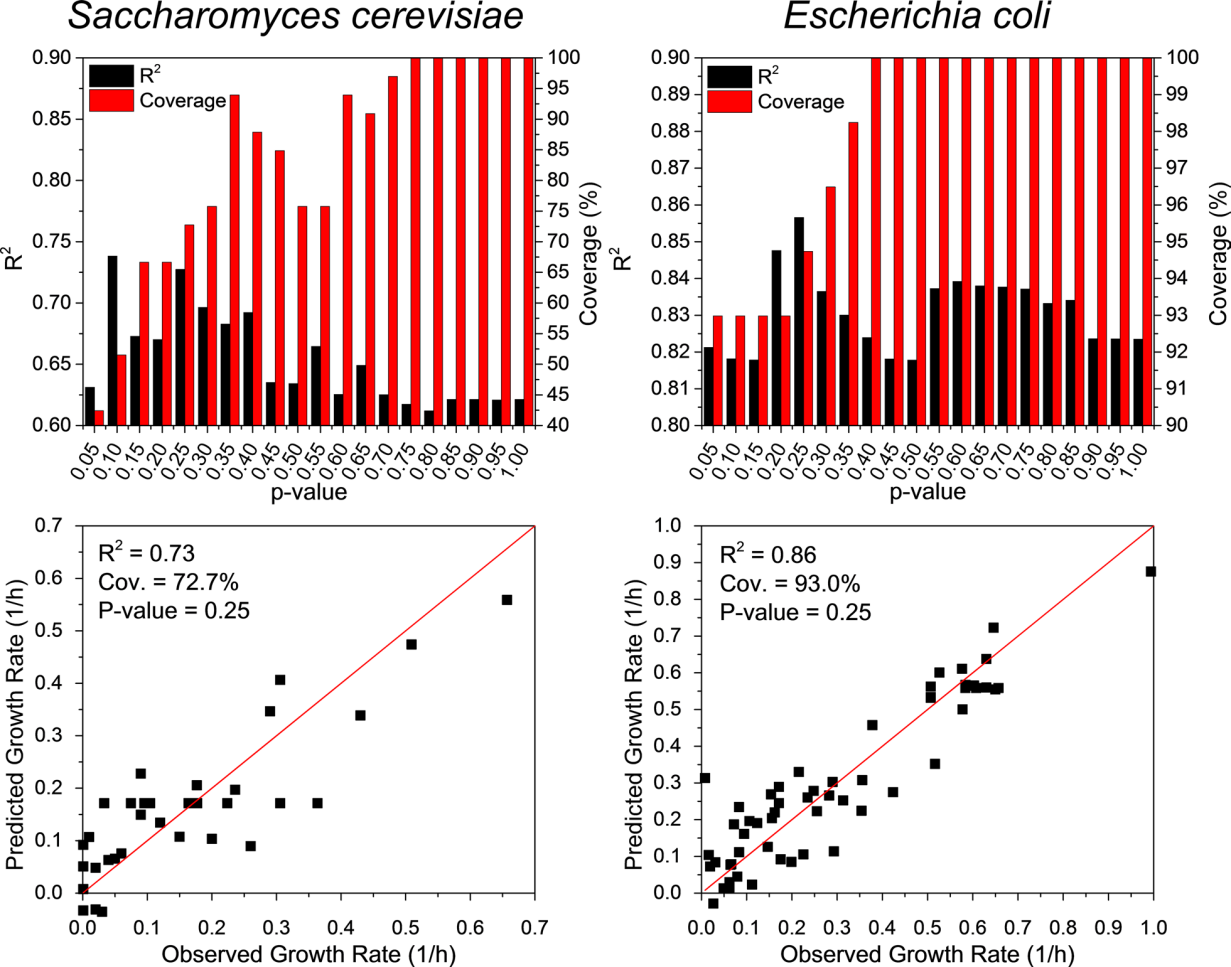
Application of the iTAP database to accurately predict growth rates of *S. cerevisiae* and *E. coli*

To explore the possibility of using iTAP to predict cell phenotype, we first calculated the Pearson’s correlation coefficient [29] of the expression levels of each gene and the corresponding cell growth rate in the entire iTAP database for *S. cerevisiae* and *E. coli*, respectively, then picked the top five genes whose expression levels were highly correlated to cell growth rate as the genetic markers for *S. cerevisiae* and *E. coli* respectively, and applied multi-variant linear regression model in MATLAB to correlate the expression levels of the genetic markers and the cell growth rates (Fig. 4). The genes whose expression levels in the test strain were not significantly different from those in the reference strain (i.e., *p* > 0.25) were set to have a fold change as zero. We found that the growth rates of both *S. cerevisiae* and *E. coli* could be accurately predicted, with R^2^ reaching 0.73 and 0.86, respectively. Also, the genetic markers we identified had high coverage of all the case studies collected in the iTAP database, reaching 72.7 and 93.0 % for *S. cerevisiae* and *E. coli* respectively. This indicated that in most of the transcriptomics studies on stress responses of *S. cerevisiae* and *E. coli*, the selected genetic markers were significantly regulated and could be generally used to predict cell phenotypes such as growth rate.

We next analyzed the effect of the *p* value, which was used to judge whether or not a gene expression level in the test strain was significantly different from that in the reference strain, on prediction accuracy of cell growth rates and the coverage of case studies in the iTAP database. We found that the prediction could maintain a high accuracy, with R^2^ ranging from 0.61 to 0.74 for *S. cerevisiae* and 0.82–0.86 for *E. coli*. With the increase of the *p*-value, the coverage of case studies in the iTAP strain increased accordingly, since the expression levels of a gene in the test strain would be more frequently recognized as significantly different from that in the reference strain with a loose threshold of the *p*-value. Overall, by using iTAP database, we successfully proved that it was indeed possible to predict cell phenotypes from the characterization of global gene expressions.

## Conclusions

In this study, the iTAP database was constructed by utilizing research papers involving stress responses for two model organisms, *E. coli* and *S. cerevisiae.* To develop iTAP database, gene expression data, specifically the *p* values and fold changes, were obtained from the GEO database, while the phenotype data was calculated from numerical information provided in multiple research papers and standardized to a defined form of representation to ensure the uniformity of the data. Till now, we have successfully curated 57 and 143 datasets for *E. coli* and *S. cerevisiae*, respectively. This study also proved that with the “big data” of coupled transcriptomics–phenotype datasets, we could achieve accurate predictions of cell phenotypes, such as growth rates, directly from transcriptomic readouts and identify the genes that affect the occurrence of the phenotype most significantly. It is intended that this open-source, web-based database will be expanded to include not only more dataseries for the existing microorganisms by considering other stress conditions, but also to increase the number of microorganisms studied and include multi-omics data in future.

## Availability and requirements

- Project name: Integrated Transcriptomics and Phenotype Database (iTAP)
- Project homepage: https://sites.google.com/a/vt.edu/biomolecular-engineering-lab/software
- Operating systems: Platform independent
- Programming language: Zoho Creator
- License: iTAP is freely available for noncommercial purposes
- Any restrictions to use by non-academics: none
